# Assessment of long-term success rates between implant-supported overdentures and conventional complete dentures: A comparative clinical study

**DOI:** 10.6026/973206300221382

**Published:** 2026-03-31

**Authors:** Ayushi Patidar, Laxman Singh Kaira, Naina Agarwal, Ruchi Patel, Kapil Laddha, Gauri Singh, Manish Jain

**Affiliations:** 1 Department of Dentistry, Government Medical College, Datia, Madhya Pradesh, India; 2 Department of Pedodontics, BBD College of Dental Sciences, BBD University, Lucknow, Uttar Pradesh, India; 3 Department of Prosthodontics and Crown and Bridge, Karnavati School of Dentistry, Ahmedabad, Gujarat, India; 4 Department of Dentistry, Monarch Dental, Euless, Texas, USA; 5 Department of Prosthodontics and Crown & Bridge, Babu Banarsi Das College of Dental Sciences, BBD University, Lucknow, India; 6 Department of Dentistry, RVRS Govt. Medical College & Attached MG Hospital, Bhilwara, Rajasthan, India

**Keywords:** Implant-supported overdentures, conventional complete dentures, long-term success, patient satisfaction, oral health-related quality of life

## Abstract

Total edentulism significantly impairs masticatory, nutritional and psychosocial health worldwide, necessitating superior prosthetic rehabilitation solutions beyond conventional complete dentures. Hence, this prospective comparative study evaluated 156 completely edentulous patients over 5 years: implant-supported mandibular overdentures with two locator attachments (n=78) versus conventional complete dentures (n=78). Implant overdentures demonstrated superior 5-year success rates (94.9% vs 76.9%, p<0.001) with acceptable marginal bone loss (1.23±0.42 mm) and consistently higher patient satisfaction scores. Oral health-related quality of life metrics remained significantly improved across all assessment periods in the implant-supported group compared to conventional dentures. Thus, we show prosthodontic standards by establishing implant-supported mandibular overdentures as the minimum treatment protocol for optimal edentulous patient outcomes.

## Background:

Complete edentulism is a debilitating oral disease that has a devastating influence on the functional, psychological and social aspects of life of a particular individual. Although there has been great improvement in preventative dentistry, the prevalence of complete loss of teeth around the world is still very high, especially in elderly groups [1]. Conventional complete dentures have also remained the main modality of treatment of edentulous patients over the last century. Nevertheless, the natural restrictions of traditional dentures, such as insufficient retention, instability, lack of masticatory capability and progressive resorption of the alveolar bone, have led to the investigation of alternative treatment methods [2]. With the advent of the osseointegrated dental implants, the treatment paradigm of the edentulous patients was changed radically. A possible solution to the problem was the introductions of implant-supported overdentures, which are more anchored by implants but still, have the features of removable prostheses that boast better retention, stability and immediate functional results [3]. Studies have always proved that mandibular two-implant overdentures are far superior compared to traditional dentures in relation to patient satisfaction and masticatory functions of the denture [4]. Implant-supported mandibular overdentures have been acknowledged by the McGill Consensus Statement and now the York Consensus Statement as the standard of care for edentulous patients [5]. Modern research has worked to measure several variables of the implant-supported overdentures, such as other types of attachment systems, the implant configurations and loading procedures. The literature has documented positive success when using implants to support overdentures with ten-year cumulative success rates of above 95% in most clinical cases [6]. These implants have a biological success that is well-reported, with the characteristics of minimal marginal bone loss and healthy peri- implant tissues [7].

It has become more and more relevant in the study of prosthodontics: patient-centred outcomes have become an essential parameter of the success of treatment and the oral health-related quality of life is becoming a very important indicator of success. Not only is a significant improvement in quality of life measures observed when using validated instruments like the Oral Health Impact Profile, but also when using implant-supported overdenture therapy, the same instrument has shown marked improvement in quality of life measures [8]. Another psychological effect of enhanced stability of the prosthesis goes further than just functional increase and encompasses social relationships, confidence in oneself and life satisfaction altogether [9]. Nevertheless, with the overwhelming evidence on the use of implant-supported overdentures, there are still several factors that guide the decisions of treatment. Some individuals may not be eligible to receive implant therapy due to economic factors; the surgery involves the complexities of the procedure, the overall systemic health of the patient and the anatomy of the place (Pierre, 2001). In addition, the fact that long-term maintenance needs and possible complications of implant-supported prostheses require selective patient selection and extensive treatment planning makes the need to select patients carefully and to plan the treatment comprehensively [11]. Most recent studies have focused on the cost-effectiveness of implant-supported compared to overdentures, both direct treatment costs and indirect benefits concerning better functioning and less frequent adjustments of the prosthetic [12]. Economic analyses indicate that even though implant-supported overdentures are more expensive to install initially, they might prove to be a cost-effective treatment where quality-adjusted life years are taken into account [13]. The development of the technology of implant surface and its prosthetic elements has led to better clinical results and a lower complication rate. The current connecting systems possess predictable retention properties and simplified maintenance procedures [14]. Nevertheless, longitudinal, comparative data of success rates of either implant-supported overdentures or conventional dentures in controlled clinical scenarios have been limited in some populations [15]. There is a significant research gap in the field of longitudinal assessments with simultaneous consideration of clinical success parameters, patient satisfaction metrics and quality of life outcomes in one cohort study. The past studies have mostly dealt with single outcome measures or a shorter follow-up, which restricts the extrapolation of results [16]. Therefore, it is of interest to critically evaluate and compare the outcomes of long-term success, patient satisfaction, oral health-related quality of life and clinical outcomes of implant-supported overdentures and conventional complete dentures in completely edentulous patients within a 5-year period of observation.

## Materials and Methods:

## Design and ethical considerations of the study:

This was a prospective comparative clinical trial undertaken at the Department of Prosthodontics, University Dental Hospital, in January 2019 and December 2024.

## Sample size calculation:

The estimation of the sample size was done on the basis of power analysis software with references to past studies that indicate some differences in the success rate of different modalities of treatment. Under the assumption that there is a 15 per cent difference in the success rates between the groups, with a power of 80 and a level of significance of 5, 68 patients per group were necessary. To consider the possibilities of patient dropouts within the five-year follow-up period, 78 patients were recruited in each of the groups, which added to a total of 156 sample participants.

## Participant selection:

Every totally edentulous patient who desired to have a prosthodontic rehabilitation was screened. Inclusion criteria included: age 50 to 75 years old, both arches of the edentulous ridge have been completely edentulous for at least six months, sufficient anterior mandible bone to place implants (as determined radiographically), physical status of American Society of Anesthesiologists classification I or II and preparedness to adhere to the study protocol and follow-up schedule. The exclusion criteria were: the patient had an uncontrolled systemic disease, received radiation therapy to the head and neck area previously, was undergoing chemotherapy, had a metabolic bone disorder, smoked heavily (more than 20 cigarettes per day), had a psychological disorder, had received bisphosphonate therapy before, poor compliance with oral hygiene and did not want to follow up.

## Group allocation:

They engaged in informed preference based on the allocation of patients to treatment groups after thorough discussions on treatment planning. Group A (Implant-Supported Overdenture Group, n=78) was mandibular implant-supported overdentures, which were retained by two implants using locator attachments in opposition to conventional maxillary complete dentures. Group B (Traditional Full Denture Group, n=78) was given conventional complete dentures in both arches made according to the normal standards of prosthodontics.

## Treatment procedures:

## Implant-supported overdenture group:

The pre-surgical planning included panoramic radiography and cone-beam computer tomography to measure the bone size and the anatomy of the body. Two endosseous implants (3.75 mm diameter and 10-13 mm length) were installed in the interforaminal area of the mandible under local anaesthetic surgery, in two stages. The implants were placed either in the canine or lateral incisor areas with sufficient inter- implant space. After three months of healing, the second stage surgery was done to insert healing abutments. Locator abutments were then made connected and mandibular overdentures were made by conventional methods of impressions. Heat-polymerised acrylic resin was used in the processing of the denture bases and locator housings, with suitable indices of retention added with the direct chairside pickup technique.

## Conventional complete denture group:

The traditional full dentures were manufactured in accordance with the principles of standardised prosthodontic protocols such as primary impressions with alginate, custom tray production, final impressions with bordering moulding and zinc oxide-eugenol paste, jaw relation records, teeth positioning based on anatomy and esthetics, wax try-in and final operations. A balanced occlusion was instituted everywhere.

## Parameters in the clinic- measured:

The major outcome measures were the success rate of the prosthetics, which was the presence of functional in situ prosthodom without replacement. There were secondary outcomes of implant survival rate (only in Group A), marginal bone level change (measured using a standardised periapical radiograph using a paralleling technique), prosthetic complications (fractures, wear of attachments, reline needs) and biological complications (peri-implant mucositis, peri-implantitis).

## Patient-reported outcome measures:

The satisfaction of patients was determined by a tested questionnaire, which included the scales of visual analogue (0-100 mm), which measured satisfaction with retention, stability, comfort, esthetics, chewing ability, speech and overall satisfaction. The Oral Health Impact Profile-14 (OHIP-14) was used to measure oral health-related quality of life, in which a low score is a sign of a high quality of life.

## Follow-up protocol:

Clinical and radiographic evaluation was done at baseline (delivery of the prosthesis) and every 1 year within 5 years. Clinical examination on the condition of the prosthesis and the function of the attachment (Group A), the health of the peri-implant tissues (Group A) and complications assessment were performed at every follow-up visit. Marginal bone level measurements on standardised periapical radiographs were taken in Group A.

## Statistical analysis:

The analysis of data was conducted with the help of the Statistical Package of Social Sciences 26.0. The means, standard deviations and percentages of all variables were computed using descriptive statistics. An independent samples t-test was applicable in cases of continuous variables, whereas a chi-square test or Fisher's exact test was applicable in cases of categorical variables. Longitudinal data analysis was done using repeated measures analysis of variance. The Kaplan-Meier survival analysis was used to approximate the prosthetic and implant survival. The level of statistical significance was defined to be p<0.05.

## Results:

A total of 156 patients completed the five-year follow-up, with no dropouts from either group. The demographic characteristics were comparable between groups, with no statistically significant differences observed (Table 1 - see PDF). Table 2 (see PDF) compares prosthetic complications between Group A (IOD, n = 78) and Group B (CCD, n = 78). The findings show a significantly higher complication rate in the CCD group across most parameters. Denture base fractures were reported in 10.3% of IOD patients compared to 30.8% of CCD patients. The difference was statistically significant (p = 0.002), indicating a substantially higher fracture incidence in the CCD group. Tooth fracture or loss occurred in 6.4% of IOD cases versus 23.1% of CCD cases. This difference was statistically significant (p = 0.004), suggesting greater structural vulnerability in conventional dentures. Relining was required in 28.2% of IOD patients compared to 66.7% of CCD patients. This highly significant difference (p < 0.001) indicates that CCDs required more frequent post-insertion adjustments due to tissue changes or instability. Attachment replacement was observed in 43.6% of IOD patients. This complication is specific to implant- supported overdentures and was not applicable (N/A) to the CCD group. While this represents a maintenance requirement unique to IODs, it does not represent a structural failure of the prosthesis. Occlusal adjustments were needed in 23.1% of IOD cases and 53.8% of CCD cases. The difference was statistically significant (p < 0.001), indicating greater occlusal instability in CCDs. Complete prosthesis remake was required in 5.1% of IOD cases versus 23.1% of CCD cases. This difference was statistically significant (p = 0.001), demonstrating higher long-term failure in conventional dentures. Soft tissue irritation was reported in 15.4% of IOD patients compared to 48.7% of CCD patients. The difference was highly significant (p < 0.001), indicating better tissue compatibility and stability with implant-supported overdentures (Table 2 - see PDF). The implant-supported overdenture group experienced fewer major complications requiring prosthesis remake compared to the conventional denture group. Biological complications in Group A included peri-implant mucositis in 14 patients (17.9%), which resolved following professional cleaning and improved oral hygiene. Peri-implantitis was diagnosed in 5 patients (6.4%) and managed successfully with non-surgical and surgical interventions. Patient satisfaction scores demonstrated consistently superior outcomes in the implant-supported overdenture group across all assessed parameters. The differences were statistically significant at all-time points and for all satisfaction domains. OHIP-14 scores demonstrated substantial improvement in oral health-related quality of life in the implant-supported overdenture group. Mean OHIP-14 scores decreased from 38.2±7.6 at baseline to 8.4±4.2 at five years in Group A, compared to a decrease from 36.8±8.2 to 24.6±8.4 in Group B. The improvement was significantly greater in Group A (p<0.001) (Table 3 - see PDF).

## Discussion:

The current research has presented abundant evidence that allows concluding that the long-term performance of implant supported relative to conventional complete dentures in full edentulous patients is better. The results show that the success rates, patient satisfaction and oral health-related quality of life in the implant-supported overdenture group are much higher and significantly better than those of the control group within a five-year observation period. The rate of success of the prosthetic used is 94.87% in the group of implant supported over denture, which is in line with the existing literature that claims positive long-term results of this treatment modality. The results of systematic reviews that have examined the outcomes of implant-supported overdentures have continued to report high survival and success rates, with ten-year outcomes reporting cumulative success rates of over 90% in most clinical practices [7]. The result of a significantly reduced success rate of the conventional denture group (76.92) can be attributed to the intrinsic inefficiency of the tissue-supported prostheses, especially in the mandible, where the ability to support the prostheses and maintain their functionality is diminished due to progressive alveolar ridge resorption [8]. The rate of 98.08% of implant survival in the current research is relatively positive as compared to the published implant literature on mandibular overdenture. Modern studies have given comparable survival rates of the implants that support mandibular overdentures and early failures are rather rare when proper selection of patients and surgical procedures are used [9]. The three cases of implant failure that were observed in the study were in patients who had controlled diabetes and this may indicate that metabolic control was the key to the success of the implant, since the recent meta-analyses that investigated diabetes as a source of implant failure reported that diabetes was a risk factor leading to implant failure [2]. Marginal bone loss patterns exhibited in the study reflected the nature of bone remodelling of the first year, followed by stabilisation, which is in line with the existing literature of peri-implant bone remodelling. The average bone loss of 1.23 -0.42 mm at five years is within the acceptable limits based on the proposed success criteria to allow up to 1.5 mm bone loss in the first year and 0.2 mm in subsequent years [10]. Such results are consistent with recent longitudinal research showing that the bone levels remain steady in the area of overdenture implant in cases where proper loading guidelines and maintenance regimes are in place [12]. The much-reduced rate of prosthetic complications in the group with the implant-supported overdenture is a clinically significant event with consequences on maintenance needs and future long-term care of treatment costs. The decreased need for relines, fewer denture base fractures and the necessity to use fewer prosthesises replacement are some of the advantages that lead to more convenient patients and possibly, better cost- effectiveness in the long run observation [13]. Nevertheless, the need to replace the component of the attachment periodically in the implant-supported overdentures must be taken into consideration when planning the treatment and counselling their patients [14]. The outcome of patient satisfaction in the present research adds to the significant amount of literature indicating a high level of satisfaction with implant-supported overdentures. The retention and stability satisfaction scores are dramatically different, which is a result of the basic mechanical benefit of the implant anchorage, which serves to deal with the major complaints of the traditional complete denture wearers [15]. Recent multicenter research has also verified that the increase in patient satisfaction after the implant-supported overdenture treatment is sustained over long follow-up periods, which substantiates the sustainability of the treatment benefits [16]. The outcomes of the OHIP-14 indicate that both groups show significant remedies to the oral health-related quality of life after the prosthodontic rehabilitation, but the fact that the increase in the quality of life is significantly higher in the group receiving implant-supported overdenture is a reflection of the enormous effects of the stability of the prostheses on the well-being of patients. These quality of life measures are not limited to functional parameters and include psychological and social aspects, with patients stating that they have greater confidence in social situations and better eating habits [17]. The clinical implications of these findings are supported by studies that showed that the oral health-related quality of life relates to general health outcomes among the ageing population [18]. The reduced prevalence of the biological complications, especially peri- implantitis (6.4%), as compared to certain published studies, could be due to the strict criteria used in selecting patients and well- organised maintenance regimes that were used in this paper. The prevention and early treatment of peri-implant diseases still constitute an imperative part of implant-supported overdenture treatment, highlighted in modern clinical practice guidelines [9]. The overall eradication of peri-implant mucositis by the intervention of the professional dental stakeholder in all the cases in question indicates the significance of frequent maintenance therapy and adherence of the patients to the oral hygiene prescriptions [3]. Weaknesses of this study are that the treatment allocation is not randomised, thus, the potential selection bias is introduced. It is possible that the preference-based group assignment had a biasing effect since patients who volunteered to be in the implant group could have been highly motivated. Also, the design is a single-centre design, which does not allow generalising the results to other clinical settings and populations. Although the five-year follow-up period offers useful data in the medium term, it fails to reflect possible longer- term complications or changes in the success rate that may be found when the follow-up is conducted over an extended period [1]. Economic consequences of treatment choice should be taken into account when making clinical decisions. Although the initial costs of implant- supported overdenture are significantly higher than those of traditional dentures, lower maintenance requirements, minimised complications and increased patient satisfaction can also play a positive role in favourable cost-effectiveness in cases of analysis over extended time horizons [2]. Future studies that include formal cost-effective studies, including quality-adjusted life year, would improve the insight into the economic aspects of treatment choice [11]. The clinical implications of such findings are that implant-supported overdentures should be recommended as the treatment option of choice for appropriate edentulous patients, as it is in line with an existing set of consensus statements. Nevertheless, traditional full dentures are still a treatment option that holds some degree of relevance in cases where patients are contraindicated to undergo implantation surgeries, unable to afford such procedures or simply do not want such operations.

## Conclusion:

Implant-supported mandibular overdentures demonstrate superior 5-year success rates (94.9% versus 76.9%), implant survival (>98%) and acceptable bone loss compared to conventional dentures in edentulous patients. Significantly higher patient satisfaction, OHIP-14 improvements and fewer complications across functional/esthetic parameters confirm implant overdentures' clinical superiority. Thus, we show implant- supported overdentures as the minimum standard of care for mandibular edentulism when clinically/economically feasible.
